# PRSS38 Is a Novel Sperm Serine Protease Involved in Human and Mouse Fertilization

**DOI:** 10.3390/ijms262311680

**Published:** 2025-12-02

**Authors:** Ania Antonella Manjon, Gustavo Luis Verón, Rosario Vitale, Georgina Stegmayer, Fernanda Gonzalez Echeverria-Raffo, Lydie Lane, Mónica Hebe Vazquez-Levin

**Affiliations:** 1 Laboratorio de Estudios de Interacción Celular en Reproducción y Cáncer, Instituto de Biología y Medicina Experimental (IBYME), Consejo Nacional de Investigaciones Científicas y Técnicas (CONICET), Vuelta de Obligado 2490, Buenos Aires C1428ADN, Argentina; aniaamanjon@gmail.com (A.A.M.);; 2 Research Institute for Signals, Systems and Computational Intelligence, sinc(i), FICH-UNL, CONICET, Ciudad Universitaria, UNL, Santa Fe S3000, Argentina; rvitale@sinc.unl.edu.ar (R.V.); gstegmayer@sinc.unl.edu.ar (G.S.); 3 Centro Médico Fertilab, Buenos Aires C1116ABI, Argentina; 4 CALIPHO Group, SIB Swiss Institute of Bioinformatics, CH-1211 Geneva, Switzerland

**Keywords:** human reproduction, fertility, peptidase, sperm, serine protease, peptidase S1 family, mouse

## Abstract

Sperm proteases are involved in several gamete interaction events leading to fertilization. This report presents a detailed analysis of the expression and localization of serine protease PRSS38 in human and in mouse spermatozoa and its involvement in fertilization-related events, using bioinformatics, cellular, biochemical, molecular, and functional approaches. Bioinformatics analyses included genomics and data analysis, prediction of protein subcellular localization and post-translational modifications, Self-Organizing Maps (SOMs) unsupervised training with other serine proteases, protein modeling (AlphaFold), and genetic variant analysis. For cellular, biochemical, and functional studies, human semen samples were obtained from healthy normozoospermic volunteers, and *cauda* epididymal sperm were collected from adult Balb-c/C57 mice. PRSS38 presence was detected in human and mouse sperm protein extracts by Western immunoblotting. Sperm PRSS38 subcellular localization was determined by fluorescence immunocytochemistry. Human sperm–oocyte interaction events were assessed by means of the mouse Cumulus Penetration Assay (CPA) using mouse COCs, the Human Hemizona Assay (HZA), and the ZP-free hamster egg Sperm Penetration Assay (SPA). Mouse sperm–oocyte interactions were evaluated by means of in vitro fertilization (IVF) with COCs and denuded oocytes. PRSS38 is proposed to be a GPI-anchored serine protease (active site: His-100, Asp-150, and Ser-245) based on bioinformatics analyses. Using commercial antibodies, protein forms of the expected Mr (human: 31 kDa; mouse: 32 and 24 kDa) were specifically immunodetected in protein sperm extracts. Immunocytochemical analysis revealed a specific PRSS38 signal in the human sperm acrosomal region, equatorial segment, and flagellum. Mouse sperm PRSS38 was immunolocalized in the equatorial segment and hook. Human sperm preincubation with specific antibodies resulted in inhibition (*p* < 0.05) of CPA, HZA, and SPA. Mouse sperm preincubation with PRSS38 antibodies impaired (*p* < 0.05) homologous IVF using COCs and denuded oocytes. Genetic variants affecting residues involved in the GPI anchor and the catalytic triad were found in individuals from the general population whose PRSS38 protease function could be altered. This study provides, for the first time, an integrated analysis of PRSS38 in human and mouse sperm, contributing to our understanding of mammalian fertilization and male infertility.

## 1. Introduction

**Infertility** affects one in six couples worldwide, with alterations in the male partner contributing to up to half of the cases, and this is expected to worsen as a result of diseases, lifestyle, and delayed parenthood, among other factors [1,2]. Successful fertilization requires a well-orchestrated sequence of events between the spermatozoa and the oocyte. After completing their morphogenesis in the testis [3], sperm undergo maturation while they transit through the epididymis, where they develop the progressive motility and ability to recognize the egg vestments (cumulus oophorus, the *zona pellucida* (ZP), and the oolemma) [4,5].

At ejaculation, sperm are deposited in the female reproductive tract, where they undergo capacitation, a process essential for acquiring full fertilizing potential. Capacitated sperm become responsive to stimuli that trigger the acrosome reaction, enabling them to release acrosomal contents, penetrate the oocyte, and eventually fuse with the oolemma. To achieve fertilization, sperm must traverse several cellular and acellular barriers. Initially, they interact with and penetrate the cumulus oophorus surrounding the oocyte. Next, they bind to the species-specific *zona pellucida* (ZP) and undergo the acrosome reaction. Sperm that have completed this reaction penetrate the ZP and bind to the oolemma, leading to membrane fusion, a process facilitated by components of the inner acrosomal membrane and the equatorial segment [6,7,8]. Despite significant advances in identifying key proteins involved in these events, many molecular players contributing to sperm function during fertilization remain unknown [9].

Among proteins involved in fertilization, serine proteases have been shown to play crucial roles at multiple stages of the reproductive process, ranging from gametogenesis to successful fertilization. Using immunological approaches, PRSS42, PRSS43, and PRSS44 were implicated in spermatogenesis [10], while PRSS50 has also been associated with germ cell development in knockout models [11]. OVCH2 has been found to contribute to sperm maturation [12], and PRSS8 to play a role in regulating sperm motility [13]. Additionally, several serine proteases, including PRSS55 [14], have been involved in sperm migration within the female reproductive tract and the achievement of fertilization. However, several mouse genes are pseudogenes in humans (i.e., PRSS42P, PRSS43P, PRSS44P, and PRSS59P). One of the most extensively studied sperm proteins, for several decades, is acrosin, a chemotrypsin-like serine protease widely distributed among mammals. Acrosin has been associated with sperm interaction with the ZP [15,16,17], and the acrosome reaction [18,19,20,21]. Alterations in acrosin have been associated with male infertility [22,23]. Recently, a homozygous variant in the acrosin gene, causing fertilization failure in a patient, was reported for the first time [24]. Interestingly, acrosin-deficient mice showed no alterations in ZP penetration [25], although they were found to have lower fertilization potential than the wild-type strain [26]. In addition to acrosin, other serine proteases have been identified in the mouse model. In particular, the serine protease PRSS21 (TESP5/testisin) was reported to play a role in sperm–oocyte interaction, specifically with the ZP [27], and the reduced fertility of Prss21-null epididymal sperm was rescued by exposure of the sperm to the uterine microenvironment [28]. Altogether, these findings underscore the functional diversity and importance of serine proteases in male fertility and the relevance of characterizing novel entities in human and mouse models.

In recent years, approaches involving transcriptomics, proteomics, and bioinformatics have been used to describe the protein repertoire of the testis and testicular germ cell lineage, but many of the detected proteins are still poorly characterized [3]. Among them, PRSS38 (also known as MPN2 or Marapsin-2), a serine protease member of the Peptidase S1 family, was listed among a large cohort of human sperm proteins detected by mass spectrometry [29], but further studies were not reported since then. Based on this background information, the present study aimed to assess PRSS38 expression, localization in human and in mouse sperm, and its involvement in fertilization-related events, using bioinformatics, cellular, biochemical, molecular, and functional approaches.

## 2. Results

### 2.1. Bioinformatics Analysis of PRSS38

**Figure 1:**
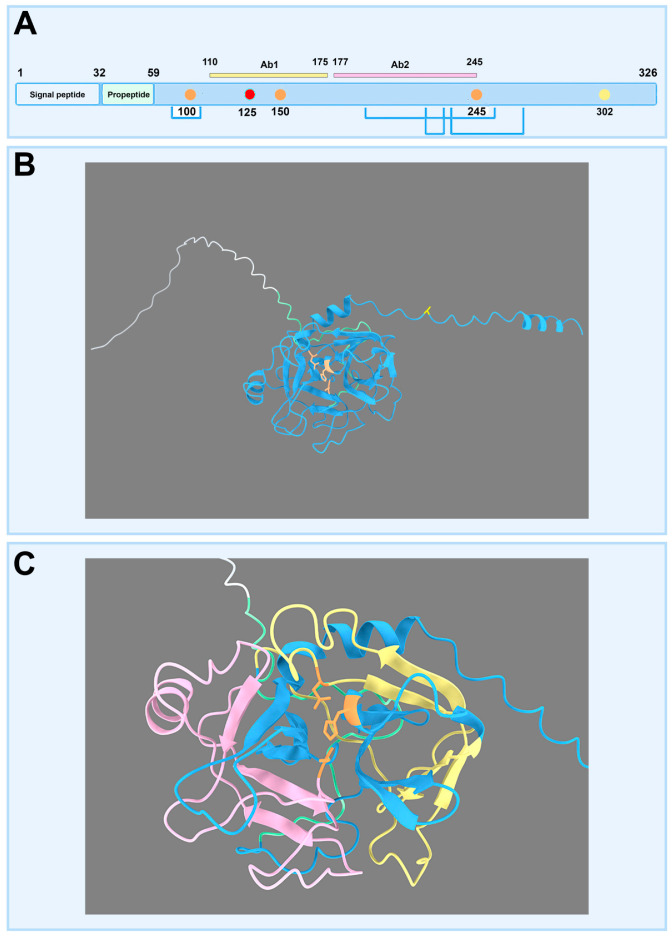
Human PRSS38 protein structure. (**A**) Linear model of PRSS38. The red dot indicates the N-glycosylation site, the yellow dot the GPI-anchor residue, the orange dots the catalytic triad, and in light blue the Cys residues involved in the disulfide bonds. Regions recognized by Ab1 (HPA055809/PA5-63186; yellow) and Ab2 (HPA028003/PA5-55748; pink) anti-PRSS38 antibodies are shown. (**B**) The tridimensional model was obtained with AlphaFold3, and the color code indicated in A. is maintained. (**C**) The tridimensional model has been zoomed in on, showing the regions recognized by the antibodies; the color code indicated in A. is maintained.

PRSS38 is a serine protease encoded on human chromosome 1 (1q42.13). This gene is transcribed to a single transcript (Ensembl code: ENST00000366757.4) that translates into the only isoform predicted (UniProtKB code: A1L453). PRSS38 has 326 amino acids, and it is predicted to be an active protease with His-100, Asp-150, and Ser-245 residues forming the active site. The PRSS38 signal peptide (residues 1–32) is followed by an N-terminal propeptide (residues 33–59). Four predicted disulfide bonds (Cys85-Cys101, Cys183-Cys-251, Cys214-Cys230, and Cys241-Cys269) and a predicted N-linked glycosylation site at Asn-125 are annotated. Although PRSS38 is classified as secreted in UniProt, PredGPI predicts a GPI anchor site in residue 302 with a 100% specificity [30]. Additional subcellular localization prediction methods based on different algorithms, such as CELLO v.2.5, WoLF PSORT, DeepLoc-2.0, and HPSLPred, also indicate protein localization to the cell membrane. Finally, pLoc-mAnimal, based on homology search by BLAST 2.7.1 and transfer of Gene Ontology (GO) annotations, predicts localization to the acrosome, but the evidence is untraceable. Taken together, these predictions lead us to propose that PRSS38 is a GPI-anchored membrane protein (Figure 1A). PRSS38 structure was confidently modeled using AlphaFold3 (pTM: 0.77), allowing for the showing of the catalytic triad (Figure 1B) as well as the peptide regions recognized by the antibodies used in the experimental assays (Figure 1C).

Since the mouse model was also utilized for experimental studies, the bioinformatics analysis of PRSS38 murine was performed. A sequence alignment between human and mouse PRSS38 proteins revealed a 57.1% identity and 82.2% similarity, with the active site and the residues involved in disulfide bonds conserved (Supplementary Figure S1). The *Prss38* gene (MGI code: 2685095) is localized in chromosome 11 and encodes a unique transcript (Ensembl code: ENSMUST00000061481.7) that translates to the only isoform predicted in the mouse (UniProt code: Q3UKY7). Mouse PRSS38 is a 322-amino-acid protein, with positions 1–28 spanning the signal peptide, and residues 29–55 the propeptide. Three N-glycosylation sites are predicted in positions 176, 250, and 276, and residues 96, 146, and 241 for the catalytic triad. Disulfide bonds are predicted in Cys residues 81–97, 179–247, 210–226, and 237–265. Finally, similar to the human counterpart, a GPI anchor site is predicted with 99.7% specificity in residue 297 by PredGPI (Supplementary Figure S2A,B). The PRSS38 murine structure was confidently predicted (pTM: 0.77), and the regions recognized by the antibodies were marked (Supplementary Figure S2C).

### 2.2. PRSS38 and Other Serine Proteases

**Figure 2:**
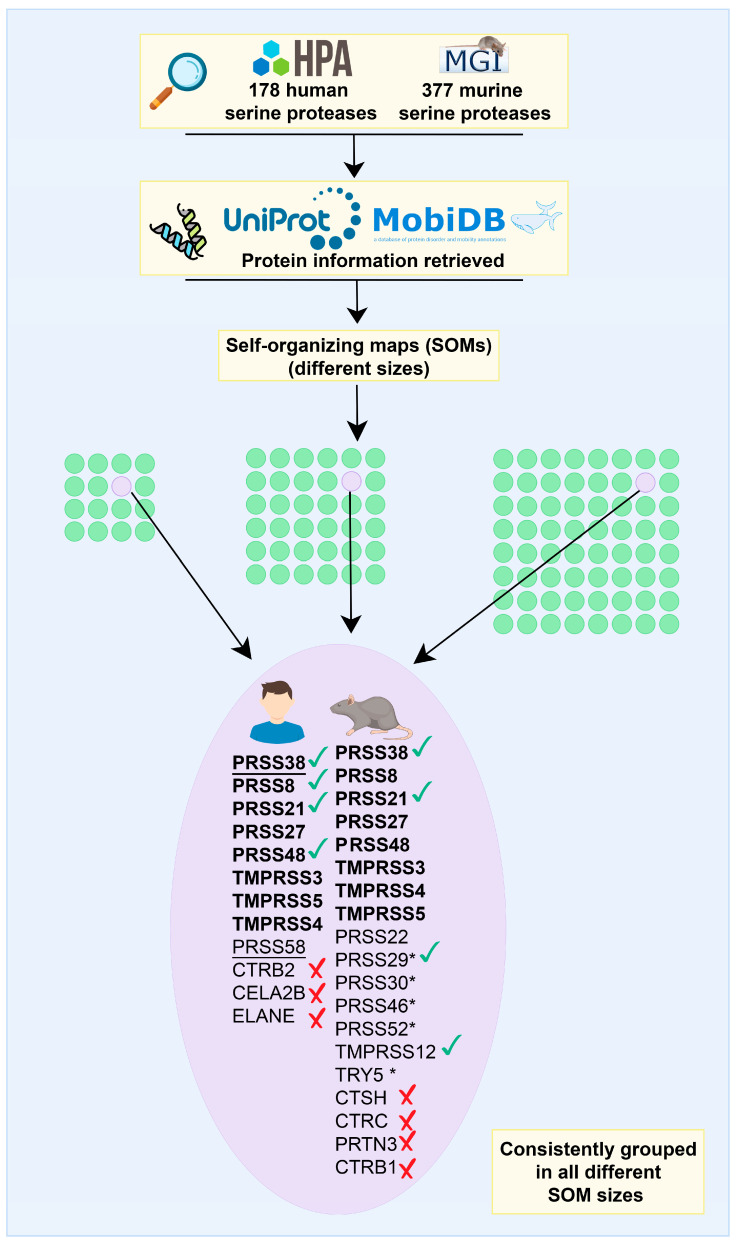
The bioinformatics machine learning pipeline developed for this study. A list of 178 human proteins and 377 murine proteins was obtained by searching the “serine protease” term in the Human Protein Atlas or Mouse Genome Informatics databases, respectively. Information for those proteins was retrieved from the UniProt and MobiDB databases and used as features in several self-organizing maps of different sizes (4 × 4, 6 × 6, and 8 × 8). Human and murine PRSS38 were always consistently grouped together with other serine proteases in all the self-organizing maps evaluated. Proteins in bold are those where human and murine counterparts were found in the neuron. Proteins with check marks were experimentally associated with roles related to fertility, while proteins with crosses are not expressed in the human reproductive tract according to the HPA or MGI. The underlined proteins are only expressed in the male reproductive tract, and the coding genes of the proteins with asterisks are pseudogenes in the human species. Flaticon (www.flaticon.com) resources were used for figure’s generation.

A list of 178 human and 377 murine proteins was obtained when the term “serine protease” was searched in the Human Protein Atlas and Mouse Genome Informatics, respectively. Features of these proteins were obtained from the UniProt and MobiDB databases and used as input in SOMs with three different sizes (4 × 4, 6 × 6, and 8 × 8 neurons). Next, the SOMs were analyzed and, as a result, 31 proteins were consistently grouped in the neuron containing human and mouse PRSS38 in all SOMs (Figure 2). Most of the listed proteins are expressed in both human and mouse testis, except for 7: human CTRB2, CELA2B, and ELANE, and mouse CTSH, CTRC, PRTN3, and CTRB1. Moreover, serine proteases already related to fertilization were found: human PRSS8, PRSS21, and PRSS48, and mouse PRSS21, PRSS29, and TMPRSS12. Interestingly, while more murine than human serine proteases were grouped in the same neuron together with PRSS38, several of the murine proteins are pseudogenes in humans, particularly PRSS29, PRSS30, PRSS46, PRSS52, and TRY5. Finally, while most of the serine proteases identified are expressed in the male reproductive tract of both species, some of them are expressed in other tissues, and all are associated with digestion (Figure 2, red crosses).

**Figure 3:**
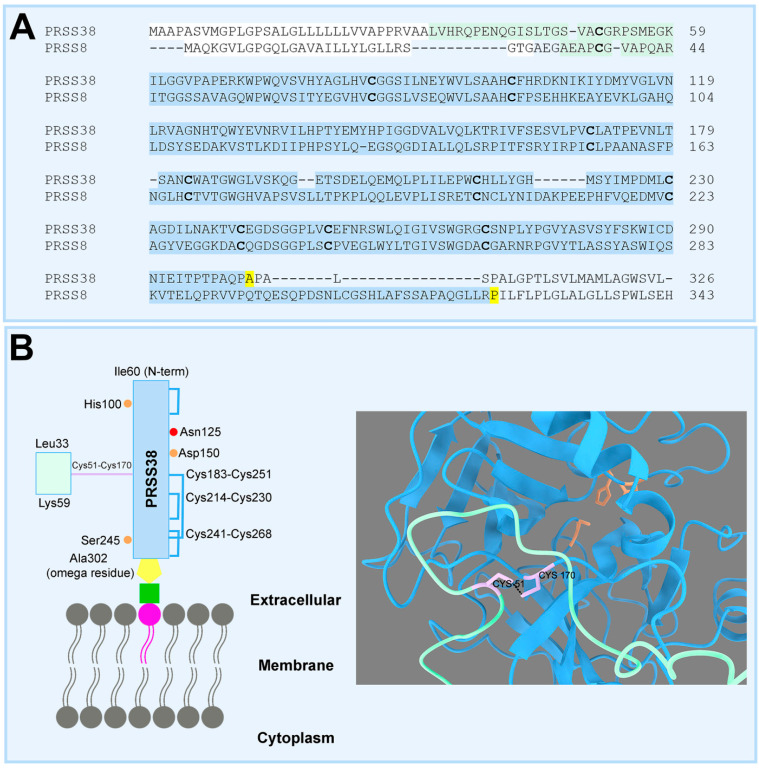
(**A**) Alignment of PRSS38 and PRSS8 amino-acid sequences. Highlighted in white is the signal peptide, in green the proposed light chain for PRSS38 and the annotated light chain for PRSS8, in blue the heavy chain, in bold the Cys residues involved in the interchain disulfide bonds, and in yellow the GPI-anchored residues (annotated for PRSS8 and predicted for PRSS38). (**B**) Proposed processing and topology for human PRSS38. By analogy to PRSS8, PRSS38 would be cleaved into a light chain (green) and a heavy chain (blue) linked by a disulfide bond and anchored to the plasma membrane by a glycosylphosphatidylinositol (GPI) molecule (yellow represents the mannose, glucosamine, and phosphoetanolamine moieties, whereas green and pink represent the phosphatidylinositol molecule).

Human PRSS38 was grouped with PRSS21 and PRSS8, both proteins previously reported as GPI-anchored to the membrane (PRSS21: [31]; PRSS8: [32]). Moreover, PRSS8 was shown to be activated by serine proteases, which cleave the pre-protein into a short light chain and a heavy chain that remain linked by disulfide bonds [33]. Instead of being processed as described in UniProtKB and neXtProt, PRSS38 could adopt a similar structure, consisting of light and heavy chains linked by a disulfide bond. In that case, part or all of the annotated propeptide may be the light chain. The Cys51 in PRSS38 would play a role analogous to the one played by Cys37 in PRSS8 (Figure 3A) and would form a disulfide bond with Cys170. The alternative model, presented in Figure 3B, is consistent with the AlphaFold model for human PRSS38 pre-protein, showing a contact with the ChimeraX analysis based on the Cys51 and Cys170 close proximity.

### 2.3. Identification of PRSS38 Genetic Variants

AlphaMissense was used for the identification of functional hotspots, defined as residues where missense mutations would generate with high probability a pathogenic change in the protein. Once the hotspot residues were retrieved (Table 1), gene variants affecting them and the catalytic triad were identified in gnomAD. Heterozygous carriers affecting most of the studied residues were found; among them, individuals carrying variants affecting His100 and Ser245 are of relevance, since these variants would affect PRSS38 protease activity. Interestingly, one XY homozygous carrier for a variant affecting Cys170, possibly involved in the light and heavy chains, was identified (Supplementary Table S1).

### 2.4. Presence and Subcellular Localization of PRSS38 in Human Sperm

Considering that PRSS38 had been detected in sperm by mass spectrometry, its presence and subcellular localization in human sperm were evaluated. To carry out these studies, commercial antibodies were used: Ab1 (HPA055809 and PA5-63186 antibodies) recognizing the same PRSS38 region (indicated in yellow), but different from the region (indicated in pink) recognized by the Ab2 (HPA028003 and PA5-55748) antibodies (Figure 1C).

**Figure 4:**
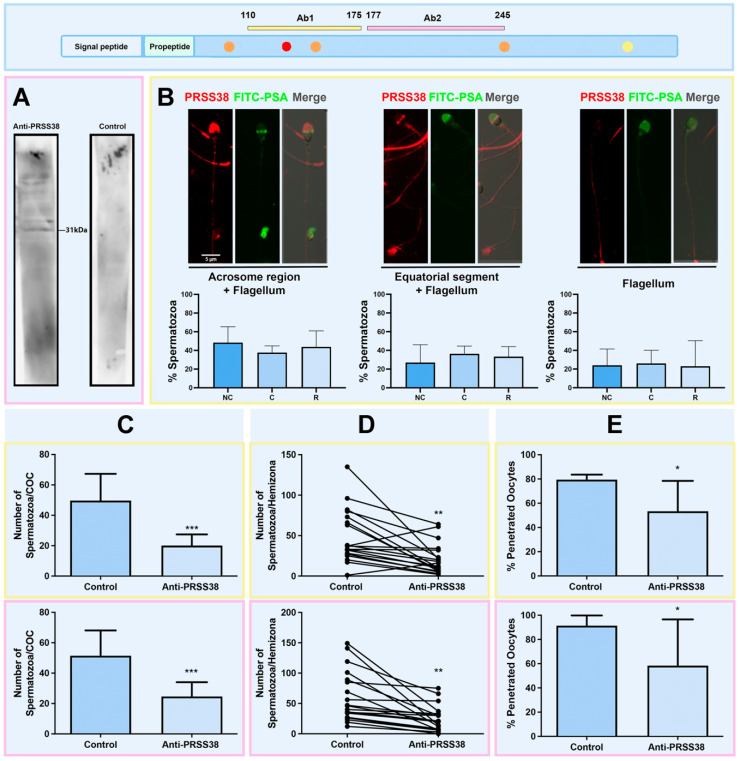
Immunodetection of PRSS38 in human spermatozoa. (**A**) Western immunoblot analysis on human spermatozoa protein extracts from a pool of three different donors using Ab2 anti-PRSS38 antibodies or purified rabbit IgG (Control) under reducing conditions. (**B**) Immunolocalization using Ab2 anti-PRSS38 antibodies. PRSS38 (red) was detected over the entire flagellum alone (F), in combination with the acrosomal region (F + A), or with the equatorial segment (F + ES). All patterns were found in acrosome-intact (non-capacitated or capacitated) and acrosome-reacted (A23187 Ca^2+^-ionophore-treated) sperm suspensions. FITC-PSA (green) staining allowed assignment of sperm acrosomal status in each cell. For each pattern, a merged image of PRSS38 and FITC-PSA is also included. Protein pattern distribution in non-capacitated, capacitated, and Ca^2+^ ionophore–acrosome-reacted spermatozoa. (**C**) Effect of anti-PRSS38 antibodies upon sperm–cumulus oophorus interaction (CPA). Human spermatozoa were pre-incubated with anti-PRSS38 Ab1 or Ab2 anti-PRSS38 antibodies or with the antibodies pre-treated with specific blocking peptides for neutralization (Control). The number of spermatozoa penetrating murine cumulus oophorus (COC) was lower after antibody incubation in both cases (*** *p* < 0.0001; Mann–Whitney test). (**D**) Effect of anti-PRSS38 antibodies upon sperm–ZP interaction (HZA). Human spermatozoa were pre-incubated with Ab1 or Ab2 anti-PRSS38 antibodies or with the antibodies pre-treated with specific blocking peptides (Control) for neutralization. The number of spermatozoa bound/penetrating the human hemizonae after antibody incubation was significantly lower in both cases (** *p* < 0.005; Wilcoxon paired test). (**E**) Effect of anti-PRSS38 antibodies on sperm–oolemma interaction (SPA). Human spermatozoa were pre-incubated with anti-PRSS38 Ab1 or Ab2 anti-PRSS38 antibodies or with the antibodies pre-treated with specific blocking peptides for neutralization (Control). The percentage of penetrated hamster oocytes was significantly lower after antibody incubation (* *p* < 0.05; Chi-squared test). HPA055809 and PA5-6318 anti-PRSS38 antibodies are indicated as Ab1 (box mark indicated in yellow), and HPA028003 and PA5-55748 antibodies are indicated as Ab2 (box mark indicated in pink).

The presence and apparent molecular weight (Mr) of PRSS38 in human sperm protein extracts from ejaculated cells freed of seminal plasma was determined by Western immunoblotting. As a result, a ~31 kDa form was immunodetected by the Ab2 anti-PRSS38 antibody; immunodetection was specific, since no protein bands were detected when the primary antibody was replaced by purified rabbit IgG (Control) added at the same concentration (Figure 4A).

Next, PRSS38 immunolocalization analysis was performed in fixed non-capacitated, capacitated, and A23187 calcium-ionophore–acrosome-reacted human sperm. Of the two antibodies available for analysis, only the Ab1 anti-PRSS38 antibody gave a positive signal in this assay. In all cases, the immunofluorescence protocol was followed by sperm staining with the FITC-PSA lectin to assign the acrosomal status in each evaluated cell. PRSS38 was immunolocalized over the entire flagellum, alone (F), and in combination with the acrosomal region (F + A) or with the equatorial segment (F + ES). In acrosome-intact non-capacitated sperm (>90% intact sperm by FITC-PSA staining), sperm stained in the acrosomal cap showed a trend more towards a higher proportion (F + A = 49.04% ± 17.26; Mean ± SDM; n = 3) than those depicting a signal in the equatorial segment (F + ES = 26.81 ± 19.09) or no signal in the head (F = 24.16% ± 17.44); however, differences were not significant amongst patterns (*p* = 0.3932; Kruskal–Wallis test). In sperm suspensions incubated in vitro for 18 h under capacitating conditions (>90% intact sperm by FITC-PSA staining), the same localization patterns were observed in acrosome-intact cells, and in comparable proportions (F + A = 37.96% ± 7.21; F + ES = 36.02 ± 8.36; F = 26.02 ± 14.16; *p* = 0.5611; Kruskal–Wallis test). Sperm suspensions incubated under capacitating conditions, followed by incubation with calcium ionophore A23187 to induce the acrosome reaction, showed a loss of the acrosome in over 50% of the cells. Acrosome-reacted sperm (FITC-PSA positive signal in the equatorial segment) depicted the same localization patterns described for intact sperm, and in similar proportions (F + A = 43.73% ± 17.25; F + ES = 33.21 ± 10.72; F = 23.07 ± 27.72; *p* = 0.3932; Kruskal–Wallis test, Figure 4B). Immunostaining was specific since no signal was observed when the primary antibody was replaced by purified rabbit IgG (Control) added at the same concentration in any of the conditions tested (Figure S3).

### 2.5. Participation of PRSS38 in Sperm–Oocyte Interaction Events

PRSS38 localization in the acrosomal region, equatorial segment, and flagellum of both acrosome-intact and acrosome-reacted sperm, along with the identification of genetic variants potentially impairing its function(s), led us to evaluate its participation in fertilization-related events. A set of in vitro assays was performed, preincubating sperm with anti-PRSS38 antibodies. For biological assays, Ab1 and Ab2 (HPA055809 and HPA028003) anti-PRSS38 antibodies that recognize different protein regions were used. In Controls, the anti-PRSS38 antibodies were neutralized by means of pre-incubating them with specific peptides immobilized in a solid phase to capture the specific immunoglobulins. Since PRSS38 was also immunolocalized in the sperm flagellum, sperm motility was monitored in biological assays using antibodies against the protein in live cells. Evaluations were performed at the end of the swim-up selection, after 18 h of capacitation, after incubation with the specific antibody or its Control (antibodies pre-treated with blocking peptides), after incubation with the nuclear dye, and at the end of the sperm–*cumulus* cell co-incubation.

#### 2.5.1. Cumulus Penetration

To evaluate the involvement of PRSS38 in human sperm–COC interaction, the heterologous CPA was performed. Sperm preincubation with anti-PRSS38 antibodies led to a decrease in the number of sperm penetrating mouse COCs when compared to Controls: Control (Ab1 + peptide) = 49.70 ± 17.61 sperm/COC (n = 30); Ab1 = 20.07 ± 7.36 (n = 30); Control (Ab2 + peptide) = 51.42 ± 16.65 sperm/COC (n = 31); Ab2 = 24.63 ± 9.38 (n = 30); *p* < 0.0001 Control versus Ab1 or Ab2 antibodies; Mann–Whitney test) (Figure 4C). Antibody inhibition was comparable in both cases: 54.7 ± 10.8% for Ab1 and 48.6 ± 7.5% for Ab2 anti-PRSS38 antibodies (*p* = 0.2222; Mann–Whitney test). Considering that sperm motility is crucial for *cumulus* mass sperm penetration, the effect of the Ab1 and Ab2 anti-PRSS38 antibodies upon sperm motility was investigated; as result, no significant changes in the percentage of motility of spermatozoa incubated with the PRSS38 antibodies when compared to Controls (final assay time Control (Ab1 + peptide) = 74.14 ± 1.92% motile sperm, Ab1 = 67.78 ± 13.98, ns; Control (Ab2 + peptide) = 61.79 ± 10.99%, Ab2 = 45.82 ± 28.00, ns).

#### 2.5.2. ZP Interaction

To assess whether PRSS38 participates in human sperm–ZP interaction, the hemizona assay (HZA) was performed with sperm pre-incubated with anti-PRSS38 antibodies. When compared to Control conditions, a decrease in the number of sperm bound/hemizona was observed when sperm were exposed to Ab1 or Ab2 antibodies: Control (Ab1 + peptide) = 48.58 ± 32.99 sperm bound/HZ (n = 19) versus Ab1 anti-PRSS38 = 20.95 ± 18.76 (n = 19); Control (Ab2 + peptide) = 60.74 ± 41.79 (n = 19) versus Ab2 anti-PRSS38 = 24.79 ± 21.27 (n = 19) (*p* < 0.005 Control versus anti-PRSS38 antibodies; Wilcoxon paired test; Figure 4D). Antibody inhibition was comparable in both cases: 62.8 ± 7.9% for Ab1 and 68.2 ± 19.1% for Ab2 anti-PRSS38 antibodies (*p* = 0.700, Mann–Whitney test). No significant changes were observed in the percentage of motile sperm at the end of the assay (final assay time Control (Ab1 + peptide) = 86.78 ± 6.63% motile sperm, Ab1 anti-PRSS38 = 40.96 ± 39.37, ns; Control (Ab2 + peptide) = 78.18 ± 4.69% motile sperm, Ab2 anti-PRSS38 = 57.60 ± 35.82, ns).

#### 2.5.3. Sperm–Oolemma Interaction

To evaluate PRSS38’s role in sperm–oolemma interaction, the ZP-free hamster oocyte assay (SPA) was done. Sperm pre-incubation with Ab1 and Ab2 anti-PRSS38 antibodies resulted in a decrease in the percentage of penetrated oocytes: Control (Ab1 + peptide) = 79.44 ± 4.19 (n = 30); Ab1 anti-PRSS38 = 53.33 ± 25.17 (n = 28); Control (Ab2 + peptide) = 91.41 ± 8.35 (n = 30); Ab2 anti-PRSS38 = 58.33 ± 38.19 (n = 29); *p* < 0.005; Chi-squared test) (Figure 4E). Although Control values differed between sets due to the experimental design, these differences were not statistically significant (91.41 ± 8.35% versus 79.44 ± 4.19; *p* = 0.47, Mann–Whitney test). Incubation with anti-PRSS38 antibodies after acrosome reaction induction with A23187 calcium-ionophore resulted in similar percentages of motile sperm (final assay time Control (Ab1 + peptide) = 55.95 ± 17.73% motile, Ab1 anti-PRSS38 = 59.26 ± 11.63, ns; Control (Ab2 + peptide) = 69.32± 18.94% motile, Ab2 anti-PRSS38 = 61.44 ± 5.44, ns).

#### 2.5.4. Progesterone-Induced Acrosome Reaction

Capacitated sperm are capable of undergoing acrosomal exocytosis when exposed to physiological stimuli, i.e., ZP and progesterone (P4) [34,35]. Several sperm acrosomal proteases have been involved in the acrosomal exocytosis and related functions [18,20,36]. PRSS38 was immunolocalized in the acrosomal region, and antibodies towards PRSS38 impaired sperm ability to fuse with hamster oocytes in the SPA (see above). Given the fact that only acrosome-reacted sperm are capable of fusing with the oocyte, the ability of anti-PRSS38 antibodies to inhibit the acrosome reaction was also tested by analyzing the effect of the anti-PRSS38 antibodies upon P4-induced acrosome reaction. Suspensions of capacitated sperm exposed to P4 showed an increase in the percentage of acrosome-reacted sperm compared to cells incubated in parallel with the vehicle (Spontaneous AR = 4.62 ± 1.33%; P4-induced AR = 10.27 ± 3.34%; *p* < 0.0001, Mann–Whitney test). Under the conditions assayed, sperm preincubation with anti-PRSS38 antibodies Ab1 or Ab2 anti-PRSS38 antibodies did not affect the response to P4: Ab1 Spontaneous AR = 6.57 ± 2.34% versus Ab1 P4-induced = 10.96 ± 6.40%, *p* < 0.0001, Mann–Whitney test; Ab2 Spontaneous AR: 4.86 ± 1.74% versus Ab2 P4-induced = 14.63 ± 3.66%, *p* < 0.005, Mann–Whitney test). In addition, the percentage of spontaneous and P4-induced AR was comparable between conditions (*p* = 0.5525 Control versus Ab1, Mann–Whitney test; *p* = 0.1957 Control versus Ab2 = 0.0606; Ab1 versus Ab2, Mann–Whitney test). Thus, under the conditions assayed, anti-PRSS38 antibodies did not impair P4-induced acrosomal exocytosis.

#### 2.5.5. Studies in the Murine Model

**Figure 5:**
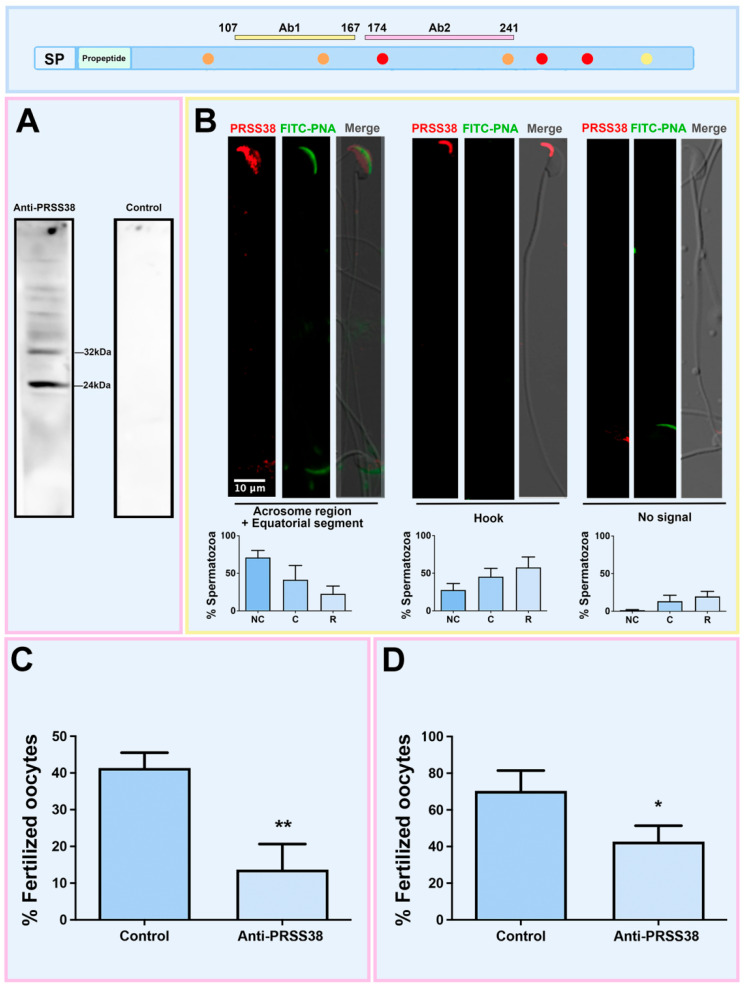
PRSS38 expression in mouse spermatozoa and its participation in homologous fertilization. (**A**) Western immunoblot analysis on *cauda* epididymal sperm protein extracts from a pool of three animals using 10 µg/mL Ab2 anti-PRSS38 antibody or purified rabbit IgG (Control). (**B**) Immunolocalization of PRSS38 in mouse spermatozoa using 4 µg/mL Ab1 anti-PRSS38 antibody. Top panel: PRSS38 (red) was detected in the equatorial segment (ES), the hook (H), or showed no detectable signal (N). All patterns were found in acrosome-intact (non-capacitated or capacitated) and acrosome-reacted (A23187 Ca^2+^ ionophore-treated) sperm suspensions. FITC-PNA (green) staining allowed assignment of sperm acrosomal status in each cell. Sperm nuclei were stained with Hoechst 33342 (blue signal). For each pattern, the merged image of PRSS38 is displayed. FITC-PNA and Hoechst 33342 are also included. Bottom panel: Pattern distribution in non-capacitated, capacitated, and Ca^2+^ ionophore–acrosome-reacted spermatozoa. (**C**,**D**) Effect of anti-PRSS38 antibodies in mouse homologous IVF (**C**: IVF with COCs; **D**: IVF with denuded oocytes). Murine spermatozoa were pre-incubated with Ab2 anti-PRSS38 antibody or with the antibody pre-treated with blocking peptides (Control) for neutralization. The percentage of fertilized oocytes was significantly lower after antibody incubation (Panel **C**, Control COCs n = 60; Anti-PRSS38 antibody COCs n = 68; Panel **D**, Control oocytes n = 32; Anti-PRSS38 antibody oocytes n = 36). * *p* < 0.05. ** *p* < 0.005; Chi-squared test. HPA055809 and PA5-6318 anti-PRSS38 antibodies are indicated as Ab1 (box mark indicated in yellow), and HPA028003 and PA5-55748 antibodies are indicated as Ab2 (box mark indicated in pink).

The murine model has been widely employed in reproductive biology to circumvent the ethical limitations associated with using heterologous oocytes to assess sperm interactions with the cumulus oophorus and binding/fusion to the oolemma. To further characterize the involvement of PRSS38 in sperm–egg interaction, expression analyses and IVF assays were performed in the murine model. The protein sequences recognized by the anti-PRSS38 antibodies are highly conserved between human and mouse proteins (Ab1 68.9% identity; Ab2 60.3% identity). Using the Ab2 anti-PRSS38 antibody for Western immunoblotting of *cauda* epididymal mouse sperm protein extracts resulted in the identification of a 32 KDa and a 24 KDa protein form (Figure 5A). Immunocytochemical localization of PRSS38 of *cauda* epididymal mouse sperm using Ab1 anti-PRSS38 antibody revealed a specific positive signal in the head, localized in the acrosomal region, equatorial segment, and hook, while some cells depicted no detectable signal for the protein (Figure 5B). Quantification of the localization patterns in non-capacitated, capacitated, and Ca^2+^ ionophore–acrosome-reacted sperm revealed the presence of all three localization patterns in the different experimental conditions. While a trend towards a predominance of the equatorial segment localization was observed in non-capacitated sperm and reached statistical significance in capacitated cells, similar proportions of all patterns were found in acrosome-reacted sperm (Figure 5B).

Based on PRSS38 localization in mouse sperm in subcellular regions involved in fertilization-related events, IVF assays with sperm pre-incubated with Ab2 PRSS38 antibody were performed as detailed in Materials and Methods. Two in vitro assays were carried out: IVF with COCs and with denuded oocytes. Results, shown in Figure 5C,D, revealed a lower fertilization rate in both assays when sperm were pre-incubated with PRSS38 antibody: % fertilized oocytes, COCs: Control = 41.33 ± 7.23 (n = 60), Ab2 Anti-PRSS38 = 13.67 ± 12.10 (n = 68), *p* < 0.005; denuded oocytes: Control = 70.33 ± 19.30 (n = 32), Ab2 Anti-PRSS38 = 42.67 ± 15.04 (n = 36), *p* < 0.05 (Chi-squared test). The inhibition rate was 76.7 ± 20.8% (COCs) and 40.0 ± 2.9% (denuded oocytes).

## 3. Discussion

Serine proteases represent a third of all proteases [37], being one of the largest peptidase groups, and several members of this group have been related to fertilization [15,38,39,40,41]. The present report characterized PRSS38, a novel sperm serine protease, using several approaches.

One of the aims of this study was to find associations between PRSS38 and other members of the vast serine protease family. While a sequence alignment analysis is capable of identifying protein paralogs, it only takes into account the sequence information. Instead, we chose to analyze all human and murine serine proteases together, considering several features, i.e., information such as the presence of a propeptide, the active site functionality, the length of the protein, and the prediction of intrinsically disordered regions. For that, different SOMs were trained due to their ability to integrate and visualize heterogeneous data through unsupervised learning [42,43]. As a result, data is clustered in the same neuron data points that share similar patterns of behavior along features [44,45].

Among the 31 proteins, a group of serine proteases already related to fertilization was found: human PRSS8, PRSS21, and PRSS48, and mouse PRSS21, PRSS29, and TMPRSS12. In particular, a soluble form of PRSS8 was also purified from human seminal fluid, suggesting that PRSS8 is synthesized in epithelial cells of the prostate gland and then secreted into the ducts [46]. In that report, it was speculated that PRSS8 could play a role during semen liquefaction that occurs a few minutes after ejaculation or during fertilization through activation of proteinases like ACR/acrosin [46]. PRSS21 was suggested to function in epididymal sperm maturation, acrosome reaction, and fertilization. In humans, PRSS21 is expressed in premeiotic germ cells [47], spermatids, and mature sperm [39]. In mice, PRSS21 is expressed in round and elongating spermatids [48] and in epididymal sperm [27,49]. The protein is also detected in luminal fluid within the *caput* and *corpus* epididymal regions, in epididymal sperm, and in epididymal epithelial cells. In addition, it was detected in the cytoplasm of testicular stallion spermatogonia and in pachytene spermatocytes but not in testicular sperm [50]. Sperm from *Prss21* knockout mice exhibit an impaired ability to undergo the acrosome reaction and to in vitro penetrate the ZP despite the normal fertility of male mice [28]. Another knockout study shows that *Prss21*-deficient mouse *cauda* epididymal sperm display low motility, abnormal morphology, increased susceptibility to head detachment, reduced ability to undergo capacitation-related phosphorylation events, and poor fertility in short-term mating studies [39]. PRSS48 was identified to be highly and specifically expressed in the testes and significantly different in non-obstructive azoospermia (NOA) patients compared with healthy Controls [51]. Finally, mouse PRSS29 has been associated with a role in hatching and implantation [52,53,54], while TMPRSS12 is involved in capacitation and acrosome reaction [55], as well as necessary for a normal motility an ZP penetration [56].

Regarding PRSS38 structure, the human and mouse proteins were modeled and predicted to be GPI-anchored to the membrane. In this sense, PRSS38 was grouped in the SOMs with GPI-anchored proteins, PRSS8 [32], and PRSS21 [32]. Netzel-Arnett and collaborators demonstrated in 2006 that PRSS8 cleaves the pre-protein into a short light chain and a heavy chain that remain linked by disulfide bonds, suggesting an alternative processing [33]. Protein similarity between PRSS8 and PRSS38, along with SOMS results and PRSS38 modeling, suggests that PRSS38 could adopt a similar structure, involving Cys51 and Cys170 in close proximity. Interestingly, a similar analysis performed in PRSS21 suggests a similar protein conformation, involving Cys33 and Cys157 residues (Figure S4). Complementary experimental studies are required to confirm these observations. Interestingly, Cys170 is considered a functional hotspot according to the AlphaMissense analysis, and an *X*Y individual with a gene variant in homozygosis affecting this residue was found in gnomAD. This variant is predicted to be pathogenic, as well as those found in carriers affecting residues of the catalytic triad of PRSS38, all of which indicate that there are individuals in the general population whose PRSS38 function may be altered and their fertility compromised. Furthermore, given that mouse models may not fully recapitulate human fertilization, there is a clear need for future genetic studies on PRSS38 to enable phenotype–genotype associations and to identify functionally relevant residues in humans.

To characterize PRSS38 expression and function, studies included human and mouse models. For this, we use two pairs of commercial antibodies targeting different segments of PRSS38 (aa 110–175 and 177–245). We identified a 31 kDa PRSS38 form in human ejaculated sperm protein extracts, the predicted molecular weight for the only isoform predicted, according to UniProt. Moreover, PRSS38 was localized in the sperm flagellum, acrosomal region, and equatorial segment of fixed sperm. This distribution remained consistent across non-capacitated, capacitated, and calcium ionophore-reacted sperm.

To further examine PRSS38 involvement in the various steps leading to fertilization, human sperm pre-incubated with specific anti-PRSS38 antibodies were subjected to various biological assays. The outcome revealed a significant reduction in sperm interaction with the cumulus oophorus, the ZP, and the oolemma upon incubation with Ab1 or Ab2 HPA anti-PRSS38 antibodies. The involvement of PRSS38 in sperm–cumulus penetration may be attributed to a direct interaction between the sperm head and the cumulus cells; this is relevant given the cumulus oophorus’ role in facilitating fertilization [57,58,59,60,61]. PRSS38 detection in the apical region of both acrosome-intact and acrosome-reacted sperm suggests its partial localization in the inner acrosomal membrane (IAM). Certain IAM proteins that remain associated with the sperm after acrosomal exocytosis have been proposed to interact with ZP glycoproteins, facilitating the progressive ZP penetration. Sperm-mediated proteolysis enables the degradation of recombinant human ZP proteins during sperm–ZP interaction [62]. Acrosin has been identified as a partner protein of other ZP-binding/penetration proteins, including Prss21, in mice [63]. As mentioned before, since PRSS38 was immunolocalized in the equatorial segment of ionophore-reacted human sperm, its role in sperm–oolemma interaction was evaluated. The human sperm penetration rate of denuded hamster oocytes was significantly reduced after sperm preincubation with anti-PRSS38 antibodies. The concept of a large multiprotein complex on both membranes forming fusion machinery has emerged in recent years [64,65]. Whether PRSS38 is part of a multiprotein complex involved in events leading to sperm binding and/or fusion to the oolemma will require further studies.

When mouse sperm protein extracts were studied, 24 kDa and 32 kDa forms were specifically immunodetected. While the latter depicted a similar Mr to the expected for the only isoform predicted in mice, the 24 kDa form may result from protein processing during sperm protein extraction, despite the inclusion of protease inhibitors in the extraction buffer. Immunocytochemical analysis of PRSS38 localization of *cauda* epididymal sperm revealed a specific positive signal in the acrosomal region, equatorial segment, and hook. While both human and murine PRSS38 share the same acrosomal region and equatorial segment localization that prompted the study of the sperm interaction with denuded oocytes, only human sperm depict a flagellar PRSS38 signal, while murine sperm show a hook signal. In this regard, both the flagellum and the hook are morphologically developed during the last phase of spermatogenesis. This event involves the temporary formation of the manchette, a transient structure that serves as a microtubular platform for nuclear shaping and flagellum formation [66]. Previous studies reported the involvement of proteases in this event, allowing tail elongation [67]. Moreover, even though the subcellular localization is different, the biological function those structures serve may be comparable, since the hook has been described to affect swimming efficiency [68]. PRSS38 participation in homologous fertilization in mice was assessed by IVF, finding significantly lower fertilization rates when sperm were pre-incubated with the anti-PRSS38 antibody. Altogether, the results, in addition to the SOMs analysis, indicate that mouse PRSS38 could have a similar role to the human counterpart, both related to fertilization.

## 4. Materials and Methods

### 4.1. Bioinformatics Tools

Gene and transcript information were retrieved from Ensembl (https://www.ensembl.org/index.html, Ensembl release 114, accessed on 25 May 2025), while protein information and alignment were obtained from UniProt (https://www.uniprot.org/, UniProt release 2025_01, accessed on 17 March 2025). Subcellular location predictions were performed with CELLO v.2.5 ([69]: http://cello.life.nctu.edu.tw/cgi/main.cgi, accessed on 20 March 2022), WoLF PSORT ([70]; https://wolfpsort.hgc.jp/, accessed on 20 November 2022), DeepLoc-2.0 ([71]; https://services.healthtech.dtu.dk/services/DeepLoc-2.0/, accessed on 15 November 2022), HPSLPred [72]; and pLocmAnimal ([73]; http://www.jci-bioinfo.cn/pLoc-mAnimal/, accessed on 20 February 2019). GPI prediction was performed with the PredGPI predictor (http://gpcr.biocomp.unibo.it/predgpi/pred.htm, accessed on 27 January 2025). Structure prediction was modeled with AlphaFold3 (https://alphafoldserver.com/, accessed on 20 March 2025), and the predicted Template Modeling (pTM) was retrieved for the model confidence evaluation, where values more than 0.5 indicated a reliable model. The models obtained were visualized with UCSF Chimera X (https://www.cgl.ucsf.edu/chimerax, version 1.8, downloaded 20 June 2024), and contacts, defined as all kinds of direct interactions (polar and non-polar), were analyzed with the corresponding tool with default features, excluding the unfavorable interactions.

### 4.2. Machine Learning Models

Self-organizing maps (SOMs) were trained to group similar serine proteases considering different features. SOMs have the ability to integrate and visualize heterogeneous data through unsupervised learning [42,43]. They can represent complex high-dimensional input patterns into a simpler two-dimensional map, where the map neurons preserve the proximity relationships of the original samples [74]. With this type of representation, it can be expected to have clustered together in the same neuron data points that share the same pattern of behavior along the features. That is, the data points grouped together in each neuron are very similar among them and very different from the other data points that belong to other neurons; thus, neurons are considered clusters. At the same time, different neurons/clusters are different from one another, uncovering hidden trends or behaviors along features. Particularly, in our analysis, all human and mouse serine proteases identified by searching “Serine protease” in the Human Protein Atlas (HPA; https://www.proteinatlas.org/, accessed on 15 April 2025) and Mouse Gene Informatics databases (MGI; https://www.informatics.jax.org/, accessed on 9 May 2025), respectively, were considered for map training. The identified proteins were searched in UniProt and MobiDB (https://mobidb.org/) databases, where features of interest were selected to consider in the SOMs. In particular, protein existence (experimental evidence at the protein level and transcript level, protein inferred from homology, protein predicted, and protein uncertain), protein length, presence of active site, binding site, and disulfide bond presence, according to UniProt, as well as disorder length and prediction of each protein according to the different predictor information, were retrieved from MobiDB (only predictors with information for at least 90% of the evaluated proteins were considered). Data was normalized prior to input into the SOM, and each feature was scaled individually to range from zero to one. Categorical features (such as protein existence) were codified with ordered numbers. In this study, instead of just selecting a determined map size, and in order to find stronger relationships among samples, several SOMs of different sizes were trained; the neuron containing the PRSS38 (human and murine) was tracked in all maps, and the serine proteases that were consistently grouped in all maps with PRSS38 were selected for further interpretation.

### 4.3. Identification of PRSS38 Hotspots and Genetic Variants

PRSS38 functional hotspots were identified using AlphaMissense, a deep learning model that integrates AlphaFold2 predictions (structural context), sequence, and population frequency, and classifies all possible genetic missense variants as probably pathogenic, probably benign, or uncertain [75]. This model is available in https://alphamissense.hegelab.org/ [76], where functional hotspots can be identified. For that, AlphaMissense calculates a pathogenicity score considering all possible substitutions for each residue; scores from 0.78 are considered highly likely pathogenic, and were the ones taken into account as hotspots in our analysis. Following the hotspot identification, gene variants affecting these and the catalytic triad residues were identified in the gnomAD database (https://gnomad.broadinstitute.org/, v4), which contains information from more than 700,000 exomes and 70,000 genomes.

### 4.4. Reagents

Analytical- and culture-grade reagents were purchased from Sigma-Aldrich (St. Louis, MO, USA), and electrophoretic reagents were from BioRad Laboratories (Hercules, CA, USA), unless otherwise specified. Anti-PRSS38 polyclonal antibodies were purchased from SIGMA (St Louis, MO, USA) (HPA028003 and HPA055809; Atlas Antibodies) and from Thermo Fisher Sci. (Carlsbad, CA, USA) (PA5-55748 and PA5-63186). Antibodies were used for immunodetection of human and mouse PRSS38 in protein extracts and whole cells, as well as in biological assays, as specifically indicated. PrEST Antigen PRSS38 peptides containing (a) the antigen sequence of HPA028003 anti-PRSS38 antibody (SIGMA), (NLTSANCWATGWGLVSKQGETSDELQEMQLPLILEPWCHLLYGHMSYIMPDMLCAGDILNAKTVCEGDS), which is the same as the antigen peptide sequence of PA5-55748 antibody (Thermo), or (b) the antigen sequence of HPA055809 anti-PRSS38 antibody (SIGMA), (IYDMYVGLVNLRVAGNHTQWYEVNRVILHPTYEMYHPIGGDVALVQLKTRIVFSESVLPVCLATPE), which is the same as the antigen peptide sequence of the PA5-63186 antibody (Thermo), were utilized in protocols designed to neutralize PRSS38 antibodies for Control experiments, as indicated along the text. Alternatively, purified rabbit IgG (SIGMA) was used in Controls. HPA055809 and PA5-6318 anti-PRSS38 antibodies are indicated as Ab1 (epitope and box marks indicated in yellow in Figure 1), and HPA028003 and PA5-55748 antibodies are indicated as Ab2 (epitope and box marks indicated in pink in Figure 1). Cy3-labeled anti-rabbit IgG secondary antibody (Chemicon-Millipore, Billerica, MA, USA) was used in fluorescence immunocytochemistry protocols and horseradish peroxidase-conjugated anti-rabbit IgG (Thermo) in Western immunoblotting assays. Hoechst 33342 (bis-benzimide; Thermo) was used to stain sperm DNA in cumulus penetration and fluorescence immunocytochemistry assays. Vectashield (H-1000) antifade solution, purchased from Vector Labs (Newark, CA, USA), was utilized to mount samples in fluorescence immunocytochemistry protocols and biological assays.

### 4.5. Protocols

All protocols were carried out in accordance with strict international biosecurity standards. Laboratory personnel were trained and periodically monitored to ensure compliance with biosecurity guidelines. Human samples (semen and oocytes) were obtained with written informed consent from donors, and all procedures were approved by the Ethics Committees of the Instituto de Biología y Medicina Experimental and Centro Médico Fertilab (Protocol #022/2017; 6 March 2018). Animals were treated in compliance with the National Institutes of Health Guide for the Care and Use of Laboratory Animals, and the study was approved by the CICUAL (Institutional Committee for the Care and Use of Laboratory Animals) of the Instituto de Biología y Medicina Experimental (IBYME) (Protocols #05/2018 (10 July 2018) and #36/2021 (6 January 2022)).

#### 4.5.1. Semen Handling

Human semen samples from 15 healthy normozoospermic volunteers were received within one hour after collection and were processed for PRSS38 analysis following standardized procedures guidelines ([77,78]). Briefly, semen samples were incubated for 15–30 min until complete liquefaction was achieved. Subsequently, semen volume, appearance, and pH were assessed, along with evaluations of sperm concentration, vitality, total and progressive motility, and morphology. Only samples with >39 million sperm per ejaculate, >58% sperm vitality, >40% motility, and >4% normal morphology (according to Kruger criteria) were included in this study.

To obtain cell suspensions enriched in non-capacitated motile human sperm (>95% motility), semen aliquots were subjected to the swim-up procedure using the Biggers, Whitten & Whittingham (BWW) medium [79] supplemented with 0.3% BSA. To promote sperm capacitation, suspensions recovered after the swim-up sperm selection technique containing 10 million sperm/mL were incubated in BWW medium supplemented with 2.6% BSA for 18 h at 37 °C in an atmosphere of 5% CO_2_ in air. In some cases, capacitated sperm were incubated with 10 μM calcium ionophore A23187 for 45 min to induce the acrosome reaction.

#### 4.5.2. Sperm Protein Analysis

Total human sperm protein extracts were prepared as previously described [80]. Semen aliquots were diluted with phosphate-buffered saline (PBS), containing benzamidine and p-amino benzamidine protease inhibitors, and centrifuged at 300× *g* for 10 min. The supernatant was discarded, and the sperm pellet was resuspended in a buffer containing PBS with 1% Triton X-100, 1% sodium deoxycholate, and a cocktail of protease inhibitors, and incubated for 2 h on ice for protein extraction. At the end of incubation, preparations were subjected to centrifugation at 10,000× *g* for 30 min at 4 °C; the supernatant containing the extracted proteins was recovered and stored at −70 °C until further analysis. Sperm protein extracts from at least three healthy normozoospermic volunteers were pooled and placed in Laemmli sample buffer under reducing conditions, boiled for 10 min, and loaded onto 15% polyacrylamide gels. Protein samples extracted from 50 million human sperm or 13 million mice sperm per lane were subjected to electrophoresis in SDS-polyacrylamide gels at 25 mA/gel constant current. Proteins were electro-transferred to a nitrocellulose membrane (Hybond-ECL; Amersham Life Biosciences, Buckinghamshire, UK) at 100 V constant for 1 h and stained with Ponceau Red solution (0.2% *w*/*v* in 0.5% acetic acid in water) to confirm adequate protein transfer and protein markers migration distance in relation to the front for molecular weight (Mr) assignment. To block non-specific sites, membranes were placed in PBS containing 0.02% Tween-20 and 5% skimmed milk (blocking buffer) for 1 h at room temperature; then, membranes were incubated overnight at 4 °C with 20 μg/mL (human) or 10 μg/mL (mouse) of Ab2 anti-PRSS38 antibodies in blocking buffer. Control experiments were performed with purified rabbit IgG, added at the same concentration as the anti-PRSS38 primary antibody. Membranes containing sperm proteins were washed to remove unbound primary antibodies, after which they were placed with HRP-conjugated anti-rabbit IgG secondary antibody (0.4 μg/mL in blocking solution) for 1 h at room temperature. Finally, membranes were washed and incubated with ECL Western blotting Detection Kit (ECL; GE Healthcare, Chicago, IL, USA), using standard procedures. Membranes were scanned with the GeneGnome XRQ-NPC Imaging system (Syngene; Heidelberg, Germany). Protein Mr was determined by comparison with molecular-weight standards (BioRad protein broad range; BioRad, Hercules, CA, USA) run in parallel.

#### 4.5.3. Immunocytochemical Sperm Analysis

To perform PRSS38 immunodetection in human sperm, cell suspensions were fixed with 2% formaldehyde in phosphate-buffered saline (PBS) for 4 min at room temperature, washed with PBS to remove the fixative, loaded onto clean slides, and air-dried. Sperm were permeabilized with methanol for 20 s at 4 °C, followed by incubation in PBS supplemented with 4% BSA for 30 min at room temperature to block non-specific sites, followed by overnight incubation at 4 °C with 2 μg/mL Ab1 anti-PRSS38 antibodies in PBS-2% BSA (purified rabbit IgG in Controls). After washing with PBS, sperm were incubated with 1 µg/mL Cy3 goat anti-rabbit IgG (H + L) in PBS at room temperature for 60 min. At the end of the immunodetection protocol, cells were incubated with 50 μg/mL fluorescein isothiocyanate (FITC) labeled *Pisum sativum* Agglutinin (PSA; SIGMA) lectin for 1 h at room temperature to assign the sperm acrosomal status. At the end of the incubation, excess lectin was removed, and sperm were mounted using Vectashield. The presence of bright staining of the acrosomal cap was indicative of an intact spermatozoon; cells showing a bright signal in the equatorial segment or lacking a signal in the acrosomal region were scored as acrosome-reacted. At least 200 sperm in each experimental condition evaluated were scored, and samples from three donors were processed in all cases. The immunostaining procedure was performed on selected post swim-up (non-capacitated), as well as those incubated under conditions to promote capacitation (capacitated), and incubated with calcium ionophore A23187 (acrosome-reacted).

To perform immunodetection of PRSS38 in mouse sperm, *cauda* epididymis from adult mice were collected, dissected, and placed in 400 μL of M16 culture medium [81] supplemented with 3 mg/mL BSA for 10 min to allow the release of motile sperm (swim-out procedure). Sperm were subjected to immunostaining (2.5 μg/mL Ab1 anti-PRSS38 antibody (or purified rabbit IgG in Controls) in PBS-2% BSA; 1 µg/mL Cy3 goat anti-rabbit IgG), followed by incubation with 50 µg/mL FITC-labeled *Peanut* Agglutining (PNA; SIGMA) lectin to assess the acrosomal status of each cell. Using this lectin, a green fluorescent signal on the acrosomal region was indicative of an intact sperm, and a lack of signal for acrosome-reacted cells. Finally, sperm were incubated with 25 µg/mL Hoechst 33342 in PBS for nuclear staining. For Controls, primary antibodies were omitted. Samples were mounted with Vectashield antifade solution and observed with an Olympus DSU IX83 confocal microscope. Micrographs were analyzed with the FIJI software (1.54f version) using the Olympus Viewer plugin [82]. At least 200 sperm were counted twice in each experiment, and three experiments were run for each evaluation reported in this study.

#### 4.5.4. Acrosome Reaction Assay

The ability of capacitated sperm to undergo acrosomal exocytosis after incubation with P4, a physiological inducer of the acrosome reaction [34], was evaluated in cells previously incubated with Ab1 or Ab2 anti-PRSS38 antibodies. Human sperm were selected by the swim-up procedure and incubated for 18 h under conditions to promote capacitation as described above. Afterwards, sperm suspensions were incubated for 1 h at 37 °C in an atmosphere of CO_2_ in air with the anti-PRSS38 antibodies; Controls were included in which the PRSS38 antibody was neutralized after preadsorption with specific peptides. At the end of the incubation, each sperm suspension was divided into two fractions, one of which was supplemented with 3 μM P4 (P8783, SIGMA) and the other with vehicle (absolute ethanol/BWW medium, 1:1), and incubated for an additional 30 min. At the end of the incubation, sperm were fixed in 2% formol and processed for acrosomal staining with FITC-PSA, as described above. The spontaneous acrosome reaction was also scored. A total of 200 sperm cells were scored in each condition, and experiments with three different donors were run. Results are expressed as the percentage of acrosome-reacted sperm.

#### 4.5.5. Sperm–Egg Interaction Assays

##### Sperm Interaction with the Cumulus Oophorus

The Sperm–*Cumulus* cell interaction assay was performed as reviewed [83]. Briefly, *cumulus* oocyte complexes (COCs) recovered from the mice oviducts 14–16 h after hCG administration were washed in culture medium and distributed in groups of 10–15 per treatment. Highly motile human spermatozoa, selected by means of the swim-up procedure, were pre-incubated for 1 h with 4 μg/mL of each specific anti-PRSS38 antibody (Ab1 or Ab2), or with the pretreated antibody to devoid specific immunoglobulins (Control condition). To achieve antibody preabsorption, the antibody was incubated for 1 h with its specific blocking peptide immobilized onto a nitrocellulose solid phase at 10 times the concentration. Then, spermatozoa were incubated with 20 μg/mL Hoechst 33342 for 10 min, after which they were added to each group of COCs and co-incubated for 15 min in capacitation medium. At the end of co-incubation, cells were washed and fixed in 2% formaldehyde for 10 min at room temperature. After washing the fixative with PBS, cells were mounted on slides and examined under a microscope equipped with epifluorescence optics (×250) to score the number of fluorescent human sperm heads within the cumulus in both experimental conditions. The assay was performed at least three times with a pool of COCS from different mice and semen samples from different donors.

##### Sperm Interaction with the ZP

Sperm interaction with the ZP was assessed by means of the HemiZona Assay (HZA) [84]. Briefly, human ZP stored in a 1.5 M ammonium sulfate solution was desalted by performing several washes in BWW medium supplemented with 0.5% BSA and split into two halves with the help of a scalpel, a procedure that included ooplasm dislodgement by pipetting. Motile sperm were selected by the swim-up procedure, and sperm concentration was adjusted to 2 million sperm/mL. A total of 50,000 sperm were placed in drops in culture plates, and pre-incubated for 1 h with each Ab1 or Ab2 anti-PRSS38 antibodies (4 μg/mL), or with the antibody pre-adsorbed with specific blocking peptides as indicated above. At the end of the pre-incubation period, hemizonae were added to the sperm drops and incubated for 4 h. At the end of the incubation, loosely bound sperm were removed by vigorous pipetting, and the number of spermatozoa tightly bound to the outer ZP surface was counted under a 400× magnification microscope using Hoffman interference optics. The HZA results were expressed as the number of spermatozoa bound per hemizona. The assay was run at least three times with a pool of oocytes from different patients and semen samples from different donors.

##### Sperm Interaction with the Oolemma

Human sperm interaction with the oolemma was evaluated with the heterologous Zona Free Hamster Egg Sperm Penetration Assay (SPA), initially described by [85]. Briefly, hamster oocytes were collected from 7- to 12-week-old female animals (*Mesocricetus auratus*) injected intraperitoneally 48 h apart with 30 IU PMSG and 30 IU hCG hormones to induce superovulation. COCs were harvested and incubated with 3 mg/mL hyaluronidase and 5 mg/mL trypsin to remove cumulus cells and ZP, respectively. Semen samples were subjected to the swim-up procedure, and motile sperm suspensions were incubated for 18 h to promote sperm capacitation, followed by 15 min incubation with 5 µM A23187 calcium ionophore to induce acrosome reaction. At the end of this procedure, sperm were washed and incubated for 30 min in BWW medium containing Ab1 or Ab2 anti-PRSS38 antibodies (4 μg/mL), or with the peptide pre-adsorbed antibody (Control). At the end of the incubations, oocytes were added to a sperm drop and incubated for an additional 2.5 h. At the end of the incubation, oocytes were washed to remove loosely bound sperm, mounted, and analyzed under phase contrast microscopy. The presence of at least one swollen head associated with a tail was indicative of successful penetration. The results on the SPA were expressed as the percentage of penetrated eggs. Assays were run with semen samples of at least three donors. The assay was run at least three times with a pool of oocytes from different animals and semen samples from different donors.

##### Murine Fertilization

To assess the impact of anti-PRSS38 antibodies on mouse sperm–oocyte interaction, in vitro fertilization (IVF) was performed in sperm previously incubated with HPA055809 anti-PRSS38 antibodies. Briefly, motile sperm were recovered from the mouse *cauda* epididymis by means of the swim-out procedure, using standard procedures. Then, sperm concentration was adjusted to 1–10 million sperm/mL in a medium supplemented with 0.3% bovine serum albumin (BSA), and cells were incubated at 37 °C in an atmosphere of 5% CO_2_ in air for a total of 90 min to promote capacitation. Anti-PRSS38 antibody (4 μg/mL) and Controls (antibodies pre-adsorbed with specific peptides as detailed above) were added to the sperm suspensions for the last 60 min of capacitation. COCs were obtained from hybrid F1 (C57Bl/6 × Balb/c) mature (2- to 6-month-old) female mice treated with an i.p. injection of equine chorionic gonadotropin (eCG; 5IU; Syntex SA, Buenos Aires, Argentina), followed by an i.p. injection of human chorionic gonadotropin (hCG; 5IU, Sigma) 48 h later. After 12–14 h, females were sacrificed, the oviducts were removed, and COCs were collected. Once the sperm incubation with Ab2 anti-PRSS38 antibody was completed, COCs were added to sperm drops, and gametes were co-incubated for an additional 3 h. At the end of the co-incubation, COCs were incubated with hyaluronidase to remove cumulus masses, fixed with 2% p-formaldehyde, and cell nuclei were stained with 20 μg/mL Hoechst 33342, mounted, and analyzed in a fluorescence microscope. Fertilization was confirmed by the presence of both male and female pronuclei (2 PN). Results were expressed as percentages of fertilized oocytes = [# of fertilized oocytes (two pronuclei; 2 PN)/# of inseminated oocytes] × 100. The assay was performed at least three times.

### 4.6. Statistical Analysis

Results are expressed as Mean ± Standard Deviation of the Mean (SDM), unless otherwise indicated. Differences in results of PRSS38 sperm (human and mouse) distribution patterns and in PRSS38 antibodies’ effect upon P4-induced acrosome reaction were evaluated using the Kruskal–Wallis test followed by Dunn’s multiple comparison test. Differences in the number of sperm penetrating murine cumulus cells or associated with human ZP were determined using the Mann–Whitney or Wilcoxon tests, respectively. Hamster oocyte penetration scores and IVF were analyzed using the Chi-squared test.

## 5. Conclusions

Altogether, findings from this report offer a comprehensive and integrative assessment of PRSS38 expression and its role in fertilization-related events in both human and mouse models. By combining bioinformatics, computational biology, and machine learning together with cellular, biochemical, and functional assays, this work advances the understanding of the molecular mechanisms underlying mammalian fertilization, providing valuable insights that may inform future studies on reproductive biology and potential clinical applications.

## Figures and Tables

**Table 1:** PRSS38 functional hotspots with a score greater than 0.78, obtained by AlphaMissense.

Residue	Position	Score
W	71	0.918
W	73	0.937
S	76	0.818
C	85	0.935
W	94	0.853
C	101	0.96
F	102	0.838
G	116	0.854
W	129	0.789
D	150	0.935
C	170	0.856
C	183	0.932
G	187	0.843
W	188	0.958
G	189	0.845
C	214	0.935
C	230	0.953
C	241	0.903
D	244	0.895
G	246	0.918
C	251	0.911
W	257	0.789
G	261	0.833
S	264	0.78
W	265	0.866
C	269	0.85

## Data Availability

The original contributions presented in this study are included in the article/Supplementary Material. Further inquiries can be directed to the corresponding author.

## Supplementary Materials

The following supporting information can be downloaded at: https://www.mdpi.com/article/10.3390/ijms262311680/s1.
