# Supplementary material for: PRSS38 Is a Novel Sperm Serine Protease Involved in Human and Mouse Fertilization
Source: Int J Mol Sci. 2025 Dec 2;26(23):11680. doi: 10.3390/ijms262311680 (PMC12691765; doi:10.3390/ijms262311680)

Supplementary Table S1. Gene variants affecting residues of interest in PRSS38 found in gnomAD

Position	Change	Variant	Allelic frequency	AlphaMissense
His 100	p.His100Pro	c.299A>C	1/402.908 (0.000001241)	LP (0.803)
Ser 245	p.Ser245Pro	c.733T>C	1/67247 (0.000007435)	LP (0.936)
Trp71	p.Trp71Arg	c.211T>C	3/806877 (0.000001859)	LP (0.972)
Trp73	p.Trp73Arg	c.217T>C	2/ 806804 (0.000001239)	LP (0.987)
Trp73	p.Trp73Leu	c.218G>T	2/806836 (6.197e-7)	LP (0.929)
Trp73	p.Trp73Cys	c.219G>T	1/806822 (6.197e-7)	LP (0.988)
Ser76	p.Ser76Arg	c.226A>C	8/806732 (0.000004958)	LP (0.984)
Ser76	p.Ser76Gly	c.226A>G	1/806791 (6.197e-7)	VUS (0.551)
Ser76	p.Ser76Asn	c.227G>A	2/807116 (6.197e-7)	LP (0.936)
Cys85	p.Cys85Gly	c.253T>G	1/806815 (6.197e-7)	LP (0.841)
Trp94	p.Trp94Arg	c.280T>C	19/806710 (0.00001178)	LP (0.962)
Cys101	p.Cys101Trp	c.303C>G	1/805362 (6.208e-7)	LP (0.979)
Phe102	p.Phe102Leu	c.306T>G	4/805337 (0.000002483)	LP (0.977)
Gly116	p.Gly116Val	c.347G>T	1/807080 (6.195e-7)	LP (0.946)
Gly116	p.Gly116Ala	c.347G>C	6/807080 (0.000003717)	LP (0.754)
Gly116	p.Gly116Asp	c.347G>A	1/807080 (6.195e-7)	LP (0.92)
Trp129	p.Trp129Cys	c.387G>T	14/807008 (0.000008674)	LP (0.936)
Cys170	p.Cys170Arg	c.508T>C	5/807024 (0.000003098)	LP (0.865)
**Cys170**	**p.Cys170Phe**	**c.509G>T**	**55/807016 (0.00003408)**	**LP (0.789)**
Cys183	p.Cys183Ser	c.548G>C	1/807100 (6.195e-7)	LP (0.961)
Gly187	p.Gly187Arg	c.559G>A	2/807100 (0.000001239)	LP (0.861)
Gly187	p.Gly187Glu	c.560G>A	1/807109 (6.195e-7)	LP (0.932)
Cys214	p.Cys214Gly	c.640T>G	3/806770 (0.000001859)	LP (0.873)
Cys230	p.Cys230Arg	c.688T>C	2/806909 (0.000001239)	LP (0.967)
Cys230	p.Cys230Tyr	c.689G>A	1/806922 (6.196e-7)	LP (0.96)
Cys241	p.Cys241Arg	c.721T>C	1/806533 (6.199e-7)	LP (0.925)
Asp244	p.Asp244Tyr	c.730G>T	2/807035 (0.000002478)	LP (0.834)

Asp244	p.Asp244Asn	c.730G>A	13/806976 (0.000008055)	LP (0.744)
Asp244	p.Asp244Val	c.731A>T	1/806893 (6.197e-7)	LP (0.931)
Gly246	p.Gly246Arg	c.736G>A	126/806991 (0.00007807)	LP (0.868)
Cys251	p.Cys251Tyr	c.752G>A	2/807038 (0.000001239)	LP (0.983)
Cys269	p.Cys269Arg	c.805T>C	1/809610 (6.195e-7)	LP (0.896)

Non-synonymous gene variants affecting residues of interest in PRSS38 found in gnomAD. All variants were found to be heterozygous except the one in bold. Scores predicting uncertain effects (VUS, variant of uncertain significance) in yellow, and pathogenic effects (P) in red. Variants affecting active site amino acids are indicated in orange.

Supplementary Figure S1. Human and mouse PRSS38 protein sequences alignment

```
Human    MAAPASVMGPLGPSALGLLLL-LLVVAPPRVAALVHRQPENQGISLTGSVACGRPSMEGK   59
Mouse    MAALTSGL-----GVLGYLLFPLLLASPTWVTSVSRRHPKSQANSLSGDVACGQPVLQGK   55

Human    ILGGVPAPERKWPWQVSVHYAGLHVCGGSILNEYWVLSAAHCFHRDKNIKIYDMYVGLVN   119
Mouse    LLGGEFARDRKWPWQVSLHYSGFHICGGSILSAYWVLSAAHCFDRGKKLETYDIYVGITN   115

Human    LRVAGNHTQWYEVNRVILHPTYEMYHPIGGDVALVQLKTRIVFSESVLPVCLATPEVNLT   179
Mouse    LEKANRHTQWFEIYQVIIHPTFQMYHPIGGDVALVQLKSAIVFSDFVLPICLPPSDLYLI   175

Human    SANCWATGWGLVSKQGETSDELQEMQLPLILEPWCHLLYGHMSYIMPDMLCAGDILNAKT   239
Mouse    NLSCWTTGWGMISPQGETGNELLEAQLPLIPRFQCQLLYGLSSYLLPEMLCAADIKTMKN   235

Human    VCEGDSGGPLVCEFNRSWLQIGIVSWGRGCSNPLYPGVYASVSYFSKWICDNIEITPTPA   299
Mouse    VCEGDSGSPLVCKQNQTWLQIGIVSWGRGCAQPLYPGVFANVSYFLSWIRYHLQIIPNPP   295

Human    QPAPALSPALGPTLSVLMAMLAGWSVL     326
Mouse    QILPSLSSSPKNTLIIFVTIMGHLLVL     322
```

Signal peptide

Propeptide

Catalytic triad conserved residue

N N-glycosilation site

C Disulfide bond cystein

Supplementary Figure S2. Mouse PRSS38 protein structure

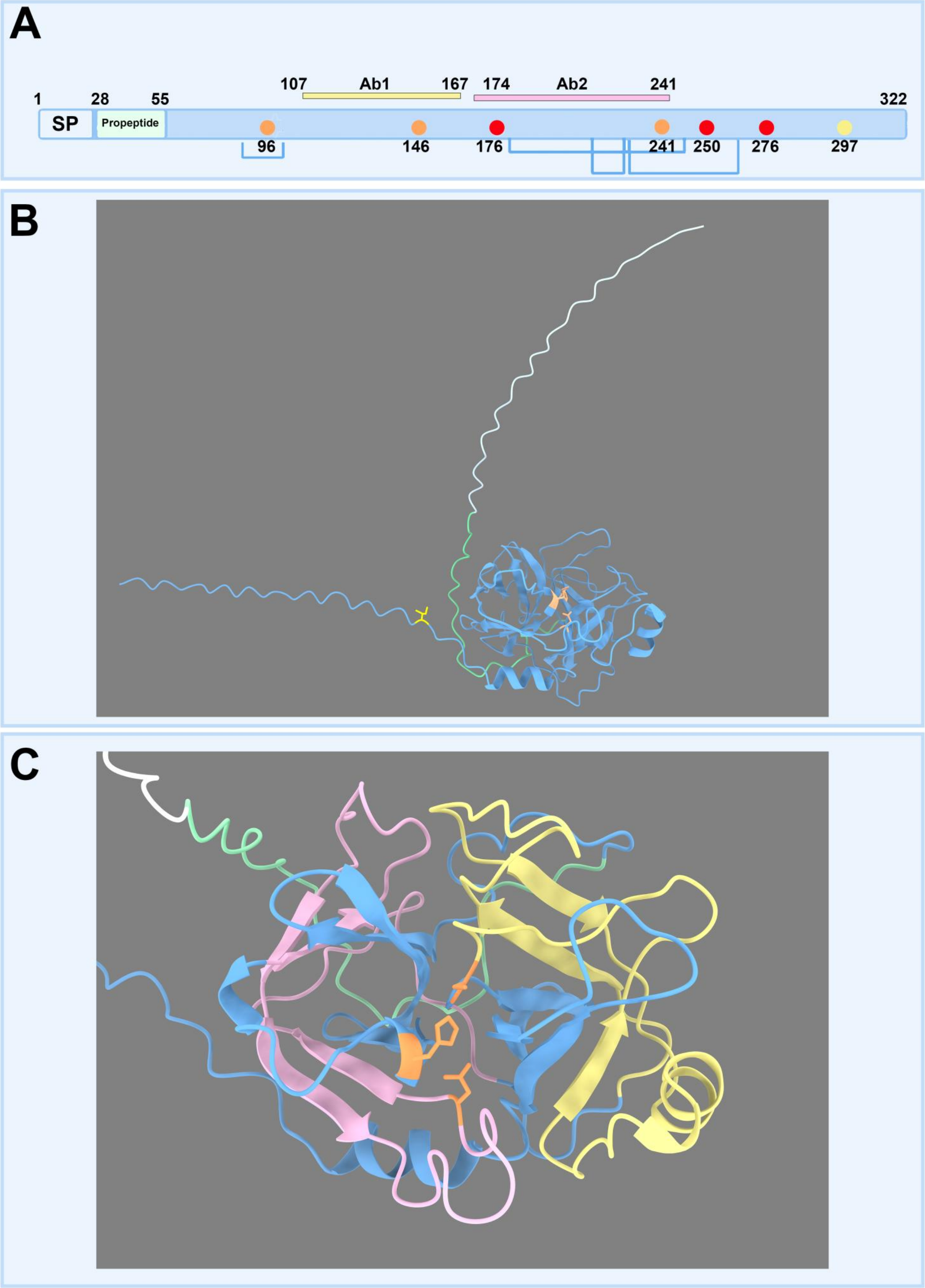

A. Linear model of mouse PRSS38. The red dot indicates the N-glycosylation site, the yellow dot the GPI-anchor residue, the orange dots the catalytic triad, and in light blue the disulfide bonds. Regions recognized by Ab1 (HPA055809/PA5-63186; yellow) and AB2 (HPA028003/PA5-55748; pink) anti-PRSS38 antibodies are shown. **B.** Tridimensional model obtained with AlphaFold3, the color code indicated in A. is maintained. **C.** Zoom in the tridimensional model, showing the regions recognized by the antibodies, the color code indicated in A. is maintained.

Supplementary Figure S3. Negative control. Immunocytochemistry of PRSS38 in Human Sperm

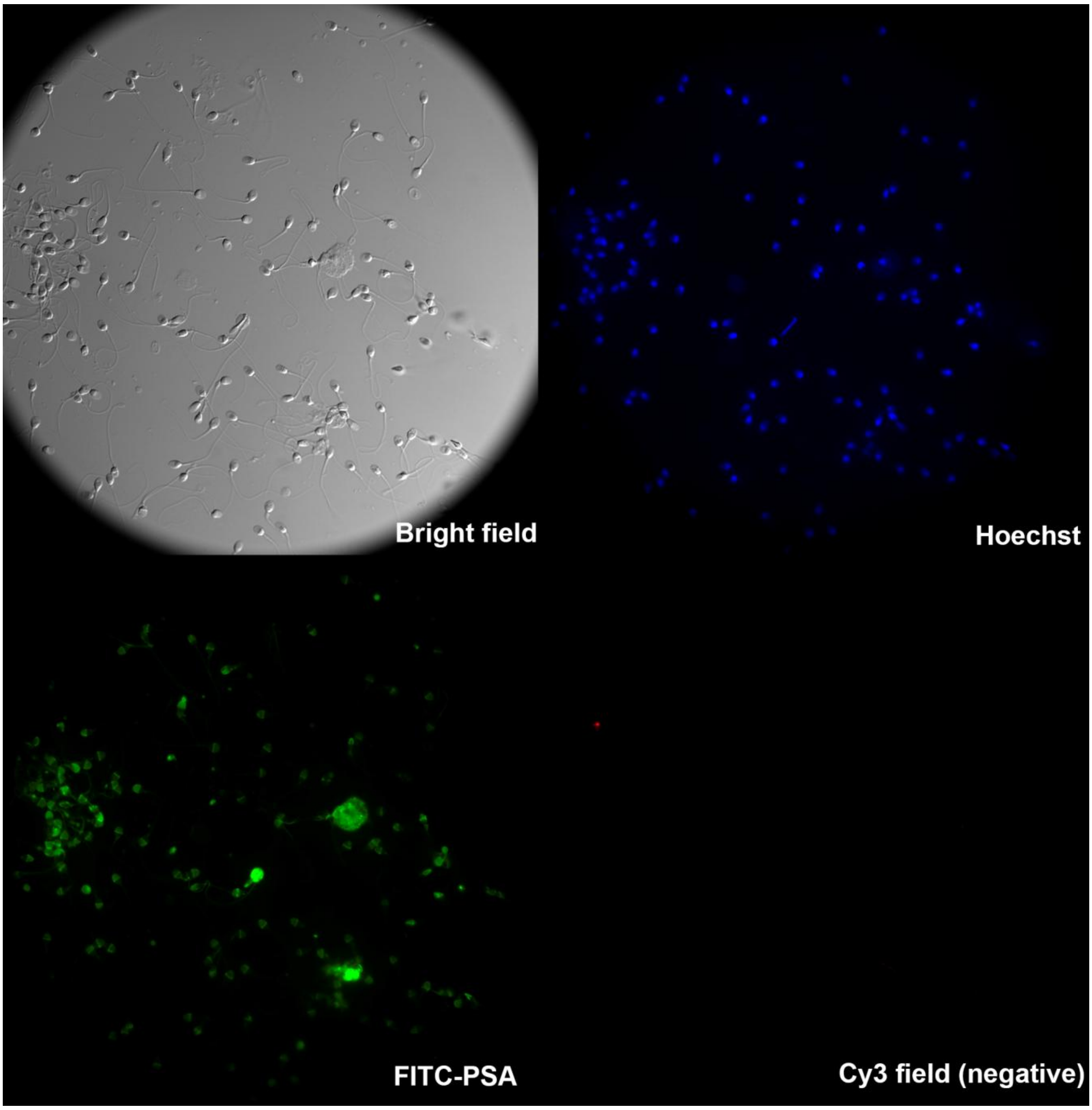

Supplementary Figure S4. Human PRSS21 structure predicted by AlphaFold3. In green the proposed light chain linked by a disulfide bond between Cys33 and Cys157 to the proposed heavy chain.

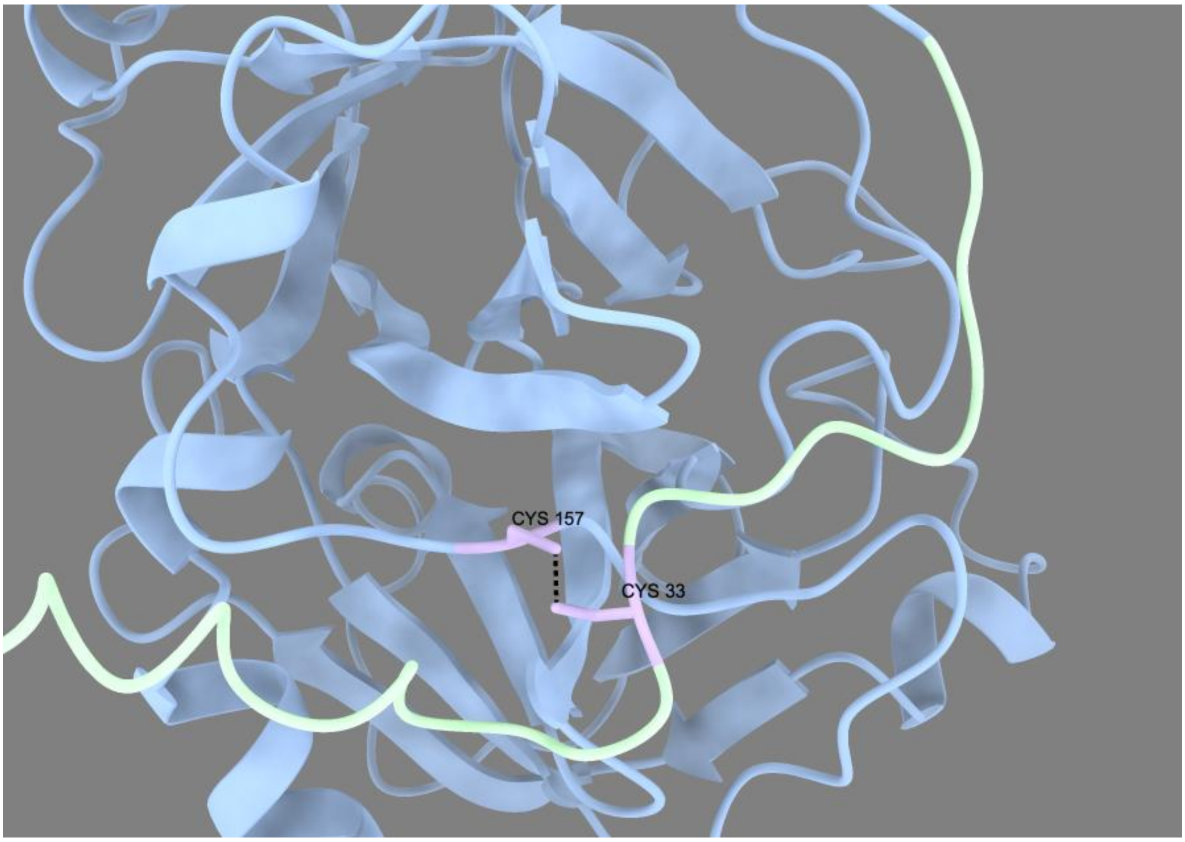

Supplement: Supplementary file 1 [file ijms-26-11680-s001.zip › ijms-3886012-supplementary.pdf]
